# A Short Study of Alcohol and Its Effects on the Human Body

**Published:** 1913-09

**Authors:** W. C. Ashworth

**Affiliations:** Greensboro, N. C.


### A SHORT STUDY OF ALCOHOL AND ITS EFFECTS ON THE HUMAN BODY.

By W. C. Ashworth, M. D., Greensboro, N. C.

Alcohol, in some of its various forms, has been so long used by man, and the literature on this subject is becoming so immense that it will be impossible for me to go very deeply into any phase of this subject, therefore, I shall ask your indulgnce if I seem to touch but lightly points that are very important. Any extended study of any prase however important would make this paper entirely too long.

All the various forms of alcohol—whiskey, brandy, wine, beer, gin and rum—have been known and used as beverages by the human race for thousands of years. Wine and beer were used to excess by the Egyptians five thousand years before Christ. Two thousand years before Christ, we learn that distilling was done in India and China. The Egyptians tombstone records tell us of many efforts to control by law, even in that early day, the evil effects of the use of alcohol. Those Pharaohs who did not drink themselves, made laws restricting the size of the vineyards; requiring the sellers of wine to be located on side streets and to take care of those who got drunk on the wine they sold. One tombstone tells us of a priest-physician who more than seven thousand years ago, built a house for the healing of the drink-madness. On other tombstones we are told of men who became habitual drunkards from blows on the head or from sunstroke.

To learn that men got drunk in those days even as they do now, we need only refer to two incidents in Genesis; one, of the curse that Noah put on Ham and his descendants, because the latter looked on the nakedness of his father when he was drunk; and the other, the unmaidenly trick that Lot's daughters played on him after they had made him drink too much wine.

The Cunieform inscriptions on the Babylonian bricks, now being dug up, tell the same story of drunkenness and the efforts to correct it. It is the same under the civilization of the Greeks and of the Romans. We find in the writings of some of their wisest lawgivers a recognition of the fact that drunkenness is a disease frequently uncontrollable and that crimes committed in that state should receive lighter punishment than ordinarily given. Their physicians recommend many remedies for its treatment.

It was a drunken horde that overran the Roman civilization and plunged Europe into the darkness of the Middle Ages. And of all those barbarions it was our ancestors the Anglo-Saxons, who had the worst reputation as gluttons and drunkards and after they came to England they kept up that reputation for more than a thousand years. And during all those years, every literature was full of drinking songs like the attractive verses of Omar Khayyam in the eighth century:

Ah my beloved fill the cup that clears
To-day of past regrets and future fears
To-morrow? Why to-morrow, I may be
Myself with yesterday's seven thousand years.

or Byron about 1815:

Fill the goblet again for I never before
Felt the glow that now gladdens my heart to its core,
Let us drink! Who would not? For in life's varied
round,
In the goblete alone no deception is found.

It is not so many generations back when it was not considered disgraceful for a gentleman to "fall under the table" at parties, public dinners, etc. And there were no warning notes given out either from the public or from the medical fraternity until comparatively recent times.

In 1745, Condillac, a French philosopher, wrote expressing clear views of the disease of inebriety, and that the state should provide means for its treatment. In 1790, our own Dr. Benjamin Rush, certainly one of the most brilliant physicians that America ever produced, expressed the same views with great clearness. From this time on, every ten or twelve years, some prominent physician in this country or in Europe would write about the evils of alcohol; but for how many years it was "as the voice of one crying in the wilderness," there were none to hear or take heed.

It was not until about 1880 that this doctrine became of such general belief that it paid chalatans to take it up and the newspapers were full of gold cures, etc., many of them being so potent that the wife could administer it in her husband's coffee without his knowledge and effect a cure. Even others were done in three days' time. In the last fifteen years we have reached the stage of scientific study when it is to be hoped that much good will be accomplished by the number who are now devoting their lives to this work.

Alcohol can be distilled from any grain containing starch and is made from potatoes, barley, rye, wheat, oats, corn and rice. The alcohol that is used medicinally and as a beverage is the second in the list of alcohols and is known as Ethyllic or grain Alcohol. ($C2H5OH$). The first alcohol is Methyllic or Wood Alcohol and is very poisonous. The third, fourth, and fifth alcohols will get mixed with the grain alcohol and are then known as fusel oils. These are more acutely poisonous than the grain alcohol itself and could be easily separated in the distilling if the distiller so desired on account of the difference in the distilling point; for the boiling point of grain alcohol is 1720F, while that of the fusel oils is 270 degrees F, 225 degrees F and 270 degrees F, respectively.

Alcohol seems to be very essential in the manufacture of drugs, chiefly as a solvent, but in my opinion, I do not think there is any place for it in the practice of medicine. If it has done good in the past as a stimulant or temporary sustainer of life, we have many drugs to take its place that will do just as well as alcohol, if not better; so that on account of the evil that it has done, I do not prescribe it at all.

Let us now inquire into the effects of alcohol on the human being, both acute and chronic. Applied locally, alcohol hardens the skin by abstractng water from it; coagulating some of it salbuminoid constituents; and dissolving its fat. It has the same effect on mucous membrane thus acting as an astringent. It is a mild antiseptic, and has some slight anaesthetic action locally perhaps by its cooling or its contracting effect on the arterioles. A single dose of alcohol introduced into the stomach has a reflex effect, making the heart beat faster and stronger and all the blood vessels dilate, especially the peripheral vessels. It produces a glow of warmth that diffuses over the whole body, and a superficial congestion of the lining of the stomach. This increased blood supply causes the stomach to throw out more gastric juice. There is a slight rise of temperature and for a short time all the bodily functions are more energetically performed.

I believe it has been agreed that alcohol in a small quantity acts as a food; that the body can dispose of an ounce or ounce and a half of pure alcohol in twenty-four hours, thus preventing the consumption of body tissue. If a considerable dose is taken the phenomena are those of exhilaration, of excitement, and of slight intoxication.

A still larger quantity (the amount differing very much with different parties, and from day to day in the same party) and the party is drunk. There is loss of muscular power, incoordination of voluntary muscles, frequent slobbering and a rambling incoherence—and then coma. With the coma there is complete muscular relaxation with stertorous breathing and as the lethal dose is approached the coma is increased. The coma from ordinary drunkenness is not profound. We can rouse the patient temporarily by loud calling, cold water to the head, etc. It is not as easy to make a certain diagnosis of drunkenness as an inexperienced person would imagine, and many a death has occurred on account of this error being made.

We will suppose a person found comatose on the street or on the public road. There is no one who knows him or can give any history of the case whatsoever. We have to diagnose drunkenness, from Apoplexy, Uraemic Coma, Coma from Opium or any other poison, Sunstroke, Epileptic Coma or from Congestion of the brain. Alcohol and cold combined will probabaly give a lower temperature than you can get from any other cause. Dr. Osler reports a temperature of 24C (75F) which had not risen over 33 degrees C (90 degrees F) in ten hours. The respiration is slow, Dr. Osler reporting a case of six per minute. In sunstroke or heat exhaustion, you invariably have fever. After epilepsy, the party will have froth on the lips, generally bloody. Perhaps the greatest difficulty will be in separating drunkenness, apoplexy, uraemic coma, opium or other poisoning. Because the breath smells of alcohol, do not jump at conclusions. It may smell of alcohol in any of these states; for a perfectly sober man may have taken a drink because he was feeling badly just before the attack came on. The breath will smell of whiskey in the drunkard perhaps of paregoric or laudanum in the opium case or of urine in the coma due to uraemia.

The pupil will have a pinpoint contraction in the opium case, dilated in the case of alcohol (Osler) and variable in apoplexy and uraemia—the two pupils may differ in both of these last. They are apt to dilate widely in all of these cases just before death. In apoplexy or head injury we can look for deviation of the eyes, paralysis of the mouth or a peculiar flacidity of the muscles of the arm or leg that would indicate paralysis. In opium poisoning and uraemia, the coma is generally more profound than in acute alcoholism. You must draw the urine and test for albumen. In uraemia, advanced so far as to cause coma, you would generally find valvular disease of the heart with arterio-sclerosis.

Having carefully and cautiously gone over your case and deciding that it is acute alcoholism alone (for you must remember that it is not impossible to have apoplexy, uraemia and acute alcoholism in the same case) you give a hypodermic of strychnine and atropine, use a stomach tube and after washing out the stomach, you give ammonia until the patient sobers up, using artificial respiration if it is necessary. Where a very large quantity of alcohol is taken at once you may have death almost instantaneously from reflex action on the heart. This is rare. Generally after an excessive dose (this differing in different individuals and the same individuals at different times) there is no stage of excitement, coma comes at once and you may have fatal convulsions.

We have brought our alcoholic patient now to the point where he can not begin his morning work until he has been properly stimulated, or rather, this applies to the steady drinker who must have about the same amount of alcohol every day, with occasional excesses, and he speer, who will go for days without touching a drop and then plunge into terrific excesses. In the steady drinker we begin to have the characteristic facies, the venules of the cheek and nose are dilated; those of the latter are enlarged and red and may produce the condition known as Acne Rosacea. The eyes are watery and the conjunctiva congested and bile-tinged. His stomach is now giving him trouble, for alcohol, at first an aid to digestion, has by overstimulation brought about a catarrhal state of the mucous membrane. The abnormal mucous, which is elaborated in great quantities, acts the part of a ferment, and starchy, saccharine, and fatty elements of the food undergo the acetic, lactic and butyric fermentations, and we have acidity, heartburn, pyrosis, and a regurgitation of food.

It will not be long before our steady drinker will be suffering from a peculiar retching in the morning known as the "morning vomiting of drunkards." But both our habitual drinker and our spreer have now begun to have "attacks." These attacks are known as Mania-a-potu or delirium tremens. A spree in a temperate person no matter how prolonged is never followed by these attacks. Few authorities make any distinction between mania-a-potu and delirium tremens, but the former should be applied to the acute, violent forms, while the latter should be applied to the chronic. Mania-a-potu is an acute attack of violent mania in which the patient must be tied or held, but after the proper remedies are administered he goes to sleep and awakens all right. What I consider the proper remedies are these: apomorphine in one-twelfth to one-eighth grain doses hypodermically, and Bromide of Soda in large doses.

In delirium tremens we have a milder form of delirium. In the habitual drinker any temporary excess may bring on an attack, or any kind of shock, or fright, an injury, especially a broken bone or disease, like pneumonia or pleurisy. Sometimes a sudden and complete withdrawal of the accustomed drink will precipitate an attack. At the outset, the patient is restless and depressed and sleeps badly. This causes him to take alcohol more freely. In a day or two the characteristic delirium sets in, the patient talks constantly and incoherently. He is always in motion and he wants to get "out of doors," in order to attend to imaginary business. Hallucinations of sight and hearing develop; he sees objects in the room such as rats, mice, snakes, monkeys, etc., and feels them crawling over his body. The terror inspired by these objects is great and has given these attacks the popular name of "the horrors." During this time, the patient should be carefully watched, for he is liable to injure himself by jumping out of a window or running away. Auditory hallucinations are not so common but do occur. He hears the roar of wild animals or the threats of imaginary enemies. It is common for him, if he hears people talking, to put entirely different words and different meanings in the mouths of those he overhears. They are abusing him, running down him character, or even plotting against his life. In the milder, more chronic forms, where the patient has had the disease repeatedly, the delusions are not necessarily attended by such great fear.

I had a patient once who thought the saw a wire sticking through the keyhole of his door; he sat down by the door and pulled imaginary wires for over two hours, talking sensibly to friends and attendants all the time and showing no signs of fright. Another was the case of an old miller, asteady drinker of many years standing who for three years before this never sat down in a room that he did not see and hear hundreds of chickens pecking corn from the floor, and this was attended by no fright or worry whatever.

In delirium tremens there is much muscular tremor; the tongue is covered with a thick white fur, and when protruded is very tremulous. The pulse is soft and rapid, readily compressed. There is fever, but it rarely goes over 103 degrees. Insomnia is constant and with utter lack of appetite. A frequent characteristic is the patient's suspicion of his friends, physicians and nurses. He imagines everything they offer him is poisoned, so it is often almost impossible to get him to take even water or whiskey. I can recall one case where this fear amounted to panophobia. He trembled near a tree or building. It would fall on him; he sat in the middle of the room; he might fall out of the window, etc. As soon as the patient sleeps nine or ten hours, he begins to improve. In more serious cases the insomnia persists, the delirium becomes incessant, the pulse more frequent and feeble, the tongue dry, the prostration extreme, with death from heart failure. The treatment for these cares varies with the symptoms. In acute mania, with a full pulse, one-twentieth to one-sixteenth grain apomorphine hypodermically will cause a rapid disappearance of the maniacal symptoms (as soon as the nausea and vomiting begin.) The patient should be placed in bed and watched night and day, for there is great danger of his jumping out of a window or running away. It is best not to tie the patient if it can be helped, but it is far better to fasten the patient to the bed with a sheet than to have three or four men holding him. At the first, you should carefully examine for any complications such as a broken bone or pneumonia.

After the apomorphine has acted, I give hypodermically morphine one-fourth grain and hyoscine one-hundredth grain every two hours until sleep is produced. I also use trional and sulfonal to produce sleep. The essential objects of treatment are to produce sleep, get the patient to eating and keep up the patient's strength. The alcohol should not be stopped entirely for several days, but about an ounce or an ounce and a half given every three hours while awake. In procuring sleep I avoid chloral and all its preparations on accounts of its depressing effect on the heart.

It is necessary to move the bowels freely from the first and to keep them moving freely. I prefer to have calomel in the first dose or two. To quiet the nerves after the first excitement is over, I use Bromide of Soda in 1-2 dr. doses. I give strychnine (one-fortieth grain) hypodermically every four hours and onurish with milk, soft boiled eggs, toast, and beef tea (Valentine's Meat Juice), and light broths. Delirium Tremens was first accurately described by Sutton of Greenwich, at the beginning of the 19th century.

As the years roll by the spreer begins to lengthen his sprees and shorten the interval between them until they overlap and he becomes a steady drinker. We have now reached the stage of the drunkard. Externally, we have a general bloated appearance, and very much enlarged venules on the face and nose, the muscular trembling, and the shambling gait with now an utter impossibility to accomplish anything in the morning until the usual amount of alcohol has been drunk. There is irritability of temper, forgetfulness, and a gradual change in the moral character. All of the mental functions are impaired, beginning first with the highest and descending to the lowest.

In intellectual centre first is impaired and then the centres that preside over animal life. The judgment also is seriously impaired, the will enfeebled, then imagination goes, then the emotions, the power of speech, muscular movements, and so on descending. The reflex centres of the cord are abolished (causing him to pass urine and faeces involuntarily) and finally the centres that control respiration and the heart. Of course, this last is a description of Dementia Paralytica, or the very last stages of drunkenness. Sometimes we have an interesting combination of symptoms known as Korsakoff's disease. There is peripheral neuritis, with loss of memory and pseudo-reminiscences—that is, false notions as to the patient's position in time and space and fabulous explanations of real occurrences. Of course, you can have this with various modifications, without the neuritis, and with only mild forms of the other symptoms.

The alcoholic is especially susceptible to germ diseases. Pneumonia is almost invariably fatal in the alcoholic. They are subject to an especially intractable form of phthisis. This is probably due to the fact that alcohol lowers the tissue vitality, thus enabling the bacteria the more readily to develop and grow. If our alcoholic dies and we perform a post-mortem, we will find every organ and tissue in the body seriously impaired. We see at once that its functions have not been half performed. From the gastric membrane to the brain tissue, the story is the same. Bartholow says he can describe the effect of alcohol in two words—fibrosis and steatosis. There is an enlargement of the connective tissue in each organ (fibrosis). This presses on the soft individual cells of that particular organ and causes their fatty degeneration (steatosis) and final destruction. In the stomach, the connective tissue cells of the mucous membrane are enlarged and hardened. They press on the cells that make the gastric juice; all are impaired, many destroyed, so that we have perverted function. In the liver, there is again growth of the connective tissue, with pressure on the true liver cells, with perverted function, there is impaired manufacture of bile, impaired storing of glycogen, etc.

In the brain and cord and nerves (in fact, in every tissue of the body) it is the same. Dr. Osler says there is more or less thickening of the pia-arachnoid membrane with wasting of the convolutions of the gray matter. In the interior of the brain, there is hyperplasia of the neuroglia with the brain cells showing fatty and atrophic changes and of course there is perverted function of the brain as described above. Alcohol seems to have an special affinity for nervous matter. It has been found in the venticles and even distilled from brain tissue, and found in greater quantity there than in any other tissue of the body.

On the average, alcohol shortens life about fifteen years—that is, those who exceed Anstie's limit of two oz. of alcohol in twenty-four hours will, on the average, shorten their lives fifteen years. Alcohol is especially liable to bring on the following diseases—gout (this is more apt to occur in England and Continental Europe than it is with us, because their wines are heavier than ours), chronic Bright's Disease, both the large white kidney and the small contracted kidney, the latter especially, for it is often called the gin drinker's arterio-sclerosis; the catarrhal condition of the stomach and indigestion, which has already been described; in old drunkards, a very intractable form of diarrhoea; Cirrhosis of the Liver and Peripherala Neuritis are diseases that can be especially claimed by alcohol. With Peripheral Neuritis, we sometimes have an optic Neuritis causing amblyopia-defective vision, with contraction of the color fields especially; Epileptoid attacks (these almost always stop when the patient gives up alcohol) and various forms of insanity.

Children inherit the alcoholic craving frequently. Many of the drunkard's children are degenerates, both physical and moral. They are especially subject to Epilepsy, Idiocy, Chorea, and Hysteria. Epilepsy, Inebriety and Hysteria are hereditary, seem to be almost interchangeable—that is—you will frequently find all three in the same family; or with Inebriety in the father, you often have Epilepsy and Hysteria in his descendants.

TREATMENT.

The RAISON D ETREE of the periodical drinker has never been found. I know of no satisfactory explanation of the peculiar symptoms of his case. After weeks, sometimes even months, of a perfectly sober life, an irresistible craving for alcohol comes on, and he immediately plunges into a terrible drinking-bout; for weeks he may pour liquor down each day to the point of coma, when suddenly there is a revulsion of feeling. He no longer craves alcohol; he pointedly dislikes it. The odor and the taste fill him with disgust. None of them seem to understand themselves. Just at this point many of them take great moral credit to themselves over this change that has occurred in them in regard to alcohol. At this time if there is a revival going on in their neighborhood, many of them will "join the church" or some temperance society. And they believe so positively that they will never take another drink they KNOW that they have reformed! And they have until the next attack of craving. It is this state of the periodical drinker that gives the quack and charlatan their oportunity. If during their treatment this attack of dislike to alcohol comes on, the drinker is convinced that the treatment has cured him and will give the strongest endorsement to gold or three days' cures, and even to that kind of cure which the wife secretly plpaces in the morning coffee.

Now is there anything that the scientific physician who wishes to make his money honestly can offer to the steady and periodic drinkers? Yes, a great deal. He can not make the man over again, but he can stop the craving for alcohol—even prevent its returning, and build him up wonderfully in a comparatively short time. The man is restored; all craving for whiskey is gone until he willfully takes his first drink. The moment he does this, the old craving comes back with all of its original force.

In the treatment of an alcoholic, the Sanitarium is almost a necessity. To have him under hourly observation; to have him away from his old associates; and best of all, to have him away from all business worries and family cares, are immense aids at the beginning of the treatment. After this we work for four things: 1st. To start his secretions; 2nds. To stop the craving (this is almost the same as the first, for to start the secretions to acting normally will frequently stop all craving); 3rd. To get him to sleeping naturally, and, 4th. To get him to eating and digesting food at regular hours. First, to start his secretions he must be purged very freely; for a week his bowels must be kept freely open. The first two nights, I like to give five grains of calomel, combined with the improved C. C. pills. This is followed early in the morning by a good dose of Pluto water or some other saline. The rest of the week his bowels are kept open by the C. C.s, alophen pills or some of the milder purgatives. Second, to stop the craving —For a week or ten days, unless there is some contra-indication, four times a day, I produce a free perspiration by the hypodermic use of Pilocarpine. The necessary dose varies from one-twelfth to one-sixth grain. I must remark in passing that this is an extremely useful drug that the average physician seems to be afraid to use. There is no danger in its use, for even if a poisonous dose is given, we have in Morphine, Atropine and Strychninen, physiological antidotes that almost immediately remove all symptoms.

Other drugs that help to remove the craving are atropine and its congeners. I give my patients a dose of a strong tonic (composed of Nux Vomica, Avena Sativa, Gentian, Vinchona, etc). Every two hours to this tonic I add a small dose of some of the mydriatics, as Atropine, Hyoscine, Hyoscamine, Scopolamine, Dubboisine, or Daturine, for all act about alike. Thous of Hyoscine I give one eight-hundredth grain at a dose, in the eight doses they take during the day. This gives them one-hundredth grain for the whole day, which is not too much. If for any reason I wish to use any of the other mydriatics, I proportion the dose in the same way. Third, to produce sleep during the first week when hypnotics are necessary, I prefer Trional or Sulphonal. You give these without danger in rather large doses. For instance, I frequently give twenty grains every hour until five or six doses are taken. I never see any bad effects the next day, save occasionally some muscular inco-ordination. The patient walks with difficulty but this passes off with the next night's sleep.

With drunkards, I never use Chloral or any of its preparations or kindred. There are two objections to these drugs. One, if your patient has any heart weakness (and drunkards so often have) you may find him dead the next morning. The other objection is that on the following day the patient is very much depressed, and this depression adds to the craving immensely. I have found Veronal a very useful drug and have seen no harm in it.

Fourth, the feeding.—I give almost as much milk as the patient will take; either sweet milk or buttermilk, the latter preferred. I use raw or boiled eggs with some cautiuonu. I do not give them until the tongue is cleaned. This caution on account of the tendency of eggs to clog the liver. I give malted milk and almost any of the starchy breakfast foods that the patient likes. I have found in cases aproaching collapse, Valentine's Meat Juice an extremely useful stimulant and food. I give it in twenty drop doses every two or three hours. The judicious administration of easily assimilated foods is of paramount importance during the convalescent state.

